# Clinical Trial Validity Guidance from the HTACG: Looking for Chicken Teeth

**DOI:** 10.3390/jmahp13020015

**Published:** 2025-04-16

**Authors:** Mondher Toumi, Bruno Falissard, Asma Jouini, Samuel Aballéa, Laurent Boyer

**Affiliations:** 1 CEReSS/UR3279—Health Services Research and Quality of Life Center, Aix-Marseille University, 13385 Marseille, France; laurent.boyer@univ-amu.fr; 2 CESP, INSERM U1018, Université Paris-Saclay, 94800 Villejuif, France; bruno.falissard@gmail.com; 3 Clever Access, Tunis 1053, Tunisia; asma.jouini@clever-access.com; 4 Inovintell, 3023 GJ Rotterdam, The Netherlands; sab@inovintell.com

The Member State Coordination Group on Health Technology Assessment (HTACG) guidance on the validity of clinical studies [1] presents an overly simplistic view of the relationship between internal and external validity in clinical trials. This approach overlooks the well-established trade-off between these two dimensions of validity, which has been extensively documented in the literature [2,3,4]. The guidance’s suggestion that internal validity, external validity, and statistical precision are independent dimensions that can all be maximized simultaneously in a single trial is problematic [1]. This view contradicts the widely accepted understanding in clinical research methodology, as illustrated by tools like PRagmatic Explanatory Continuum Indicator Summary-2 (PRECIS-2) [5,6], which conceptualizes trials along a continuum from explanatory (high internal validity) to pragmatic (high external validity).

Several factors may have contributed to this misunderstanding:

1. The epistemological debate on the internal–external validity trade-off or prerequisite [2,3,4].
2. A narrow conceptualization of external validity [7].
3. The misinterpretation of internal validity as restricted to the absence of systematic error, which only represents one facet (although critical) of internal validity [3,8,9,10].

The guidance’s approach fails to acknowledge the reality of clinical trial design, where researchers must often sacrifice external validity to achieve high internal validity [2,6,11,12], particularly in regulatory trials. This is evident in the high exclusion rates seen in many randomized controlled trials (RCTs), which can significantly limit their generalizability. For example, clinical trials in asthma exclude 95% of patients [13], while trials in Chronic Obstructive Pulmonary Disease (COPD) exclude 83% [14]. A large-scale analysis of external validity, covering 43,895 trials and 5,685,738 individuals across 989 unique drugs and 286 conditions, found that the median RCT population exclusion proportion was 81.5%, leaving RCTs with very low external validity [15]. Moreover, the HTACG guidance overlooks more nuanced approaches to balancing internal and external validity, such as the estimand framework recently adopted by the European Medicines Agency (EMA) [16]. This framework offers a more sophisticated way to reinforce internal validity and address external validity concerns in trial design and analysis, particularly by considering intercurrent events from different perspectives. However, it was only briefly mentioned in the guidance on multiplicity issues without reference to external validity [17].

The absence of discussion on pragmatic trials and estimands in the guidance is surprising, especially given the historical contributions of French academics to the field of pragmatic trials [18], while less surprising for estimand, given the German resistance to integrating estimand in the HTA framework [19]. A more comprehensive literature review, as recommended by evidence-based medicine principles referenced in the EU-HTA regulation, could have helped avoid these oversimplifications and provided a more accurate representation of the complexities involved in assessing clinical trial internal and external validity [20,21].

A review of the guidance is needed to

1. Acknowledge the need to balance internal and external validity.
2. Ensure that the estimand framework enhances external validity, given regulators’ preference for internal validity in new interventions.
3. Require the use of PRECIS-2 to assess the balance between internal and external validity.
4. Introduce pragmatic trials and discuss their associated uncertainty due to lower internal validity [22].
5. Address adaptive design trials, as they allow starting with a very narrow population with high internal validity and expanding to a broader population, thus enhancing external validity [23].

## Abbreviations

The following abbreviations are used in this manuscript:

COPD	Chronic Obstructive Pulmonary Disease
EMA	European Medicines Agency
EU-HTA	European Health Technology Assessment
HTA	Health Technology Assessment
HTACG	Member State Coordination Group on Health Technology Assessment
PRECIS	PRagmatic Explanatory Continuum Indicator Summary
RCT	Randomized Controlled Trial
